# Improved Transgenic Mouse Model for Studying HLA Class I Antigen Presentation

**DOI:** 10.1038/srep33612

**Published:** 2016-09-16

**Authors:** Man Huang, Wei Zhang, Jie Guo, Xundong Wei, Krung Phiwpan, Jianhua Zhang, Xuyu Zhou

**Affiliations:** 1 CAS Key Laboratory of Pathogenic Microbiology and Immunology, Institute of Microbiology, Chinese Academy of Sciences (CAS), Beijing, 100101, China; 2 University of Chinese Academy of Sciences, Beijing, China; 3 University of Phayao 19 Moo 2 Maeka, Muang Phayao district, Phayao, 56000, Thailand; 4 Savaid Medical School, University of Chinese Academy of Sciences, Beijing 101408, China

## Abstract

HLA class I (HLA-I) transgenic mice have proven to be useful models for studying human MHC-related immune responses over the last two decades. However, differences in the processing and presentation machinery between humans and mice may have profound effects on HLA-I restricted antigen presentation. In this study, we generated a novel human TAP-LMP (hTAP-LMP) gene cluster transgenic mouse model carrying an intact human TAP complex and two human immunoproteasome LMP subunits, PSMB8/PSMB9. By crossing the hTAP-LMP strain with different HLA-I transgenic mice, we found that the expression levels of human HLA-I molecules, especially the A3 supertype members (*e.g.*, A11 and A33), were remarkably enhanced in corresponding HLA-I/hTAP-LMP transgenic mice. Moreover, we found that humanized processing and presentation machinery increased antigen presentation of HLA-A11-restricted epitopes and promoted the rapid reduction of hepatitis B virus (HBV) infection in HLA-A11/hTAP-LMP mice. Together, our study highlights that HLA-I/hTAP-LMP mice are an improved model for studying antigen presentation of HLA-I molecules and their related CTL responses.

Human leukocyte antigen class I (HLA-I) molecules that present antigenic peptides to CD8^+^ T cells and trigger the cytotoxic T lymphocyte (CTL) response are essential for the human immune system to combat viral infections and clear transformed tumor cells^1^,^2^. A better understanding of HLA-I-related antigen processing and presentation would improve the rational design of preventive or therapeutic vaccines against viral infection and cancer. Early studies using HLA-I transfected into murine cells^3^,^4^, as well as later efforts utilizing HLA-I transgenic mice^5^,^6^,^7^, have provided a wealth of knowledge about the antigen presentation of different HLA-I molecules. However, existing differences between humans and HLA-I transgenic mice make it difficult to determine whether responses in the transgenic mice exactly reflect responses in humans^8^. Distinctions between the mouse and human antigen processing and presentation machinery may account for some of these differences^8^.

In both humans and mice, the MHC class I antigen presentation pathway proceeds through several stages^9^,^10^: i) endogenous proteins in the cytosol are degraded into short peptides of 3–22 residues by the proteasome or immunoproteasome; ii) peptide products of 8–12 amino acids are transported into the endoplasmic reticulum (ER) by a dimer complex, the transporter associated with antigen processing (TAP); and iii) empty MHC class I molecules in the ER are stabilized by binding to suitable peptides to form peptide-MHC complexes, which are then exported to the cell surface for presentation to CD8^+^ T cells. The TAP complex, which is composed of TAP1 and TAP2, is essential for peptide transportation into the ER, where the peptides bind to MHC class I molecules. Deficiency of TAP1 and/or TAP2 in mice and humans results in a severe defects in MHC class I antigen presentation and a substantial reduction in CD8^+^ T-cell numbers^11^,^12^,^13^,^14^,^15^.

Previous studies of human or animal TAP transportation demonstrate that the TAP complex selects peptides with preferential sequences, and TAP binding affinity has a significant impact on epitope presentation^16^,^17^,^18^,^19^,^20^,^21^. A minimal TAP affinity is required for peptide presentation. Epitopes with high TAP affinity can easily to be selected and recognized by CTLs. Interestingly, the peptide binding specificities between human and mouse TAP are quite different. The murine TAP displays strong specificity for binding to peptides with hydrophobic C-termini, and the human TAP demonstrates a more permissive preference in binding to peptides with both hydrophobic and basic termini^20^. Aside from differences in TAP specificity, the immunoproteasome and TAP binding protein (tapasin) also display differences between humans and mice^22^,^23^.

HLA molecules are extremely polymorphic; >10,000 alleles have been found to date^24^. Nine major HLA-I supertypes are classified according to their structural similarities in the antigen binding groove and overlapping peptide binding properties^25^. Among these supertypes, B7, A3, A2, A24, B44, A1, and B27 are the most prevalent in different ethnic groups^25^. Interestingly, HLA-A3, A24, and B27 binding peptides have higher affinity for human TAP than that of HLA-A2 and B7^26^, supporting the notion that particular HLA-I molecules co-evolved with TAP for efficient peptide processing and presentation.

The A3 supertype alleles are found in all major ethnic groups worldwide, with an average frequency of 44.2%, whereas a higher frequency of 52.7% is found in the Chinese population^25^. Alleles of this supertype, including HLA-A*0301, A*1101, A*3101, A*3301, and A*6801, share a specificity for binding to peptides with small or aliphatic residues at position 2 and basic residues at the C-terminus^27^. In accordance with this property, antigen presentation of A3 supertype alleles is more dependent on human TAP than alleles of other supertypes^28^. A representative mouse model, the HLA-A11 transgenic mouse, has been generated and used to study HLA-A11 antigen presentation and related CTL responses^5^. Although A11-restricted CTL responses are elicited following direct peptide immunization, these animals fail to generate adequate HLA-A11-restricted CTL responses during influenza virus infection^5^. Another study also supports the same notion that HLA-A11 transgenic mice have low efficiency in transporting HLA-A11-restricted epitopes, likely due to lack of human TAP^29^.

The TAP1 and TAP2 genes are tightly linked to the genes encoding the two catalytic subunits of the immunoproteasome, PSMB9 (LMP2) and PSMB8 (LMP7), which preferentially create antigenic peptides^30^. The close linkage of the TAP-LMP genes is highly conserved, being found in almost all species of vertebrates examined. Interestingly, TAP-LMP is also closely associated with MHC-I in ectothermic vertebrates such as bony fish, sharks, and amphibians, forming a compact class I unit. However, in most mammals, the TAP-LMP gene cluster is separated from class I genes, located in the class II region, which likely allowed for a more permissive evolution^30^.

Here, we generated a novel human TAP-LMP transgenic mouse (hTAP-LMP) by microinjection of the 180-kb human BAC (bacterial artificial chromosome) RP11–10A19, which carries the genes encoding intact TAP-LMP (TAP1, TAP2, PSMB8, and PSMB9). The resultant mice were crossed to various HLA-I transgenic mice to evaluate the effects of human TAP-LMP on HLA-I antigen presentation and HLA-I-restricted CTL responses. We found that the expression levels of human HLA-I, especially the A3 supertype, and the HLA-A11-restricted CTL response were notably enhanced by expression of the humanized processing and presenting machinery. Our study highlights the notion that particular HLA-I molecules co-evolved with hTAP-LMP for efficient peptide processing and presentation and emphasizes that HLA-I/hTAP-LMP mice are improved models for studying antigen presentation of HLA-I molecules and their related immune responses.

## Results

### Generation of hTAP-LMP transgenic mice

**Figure 1:**
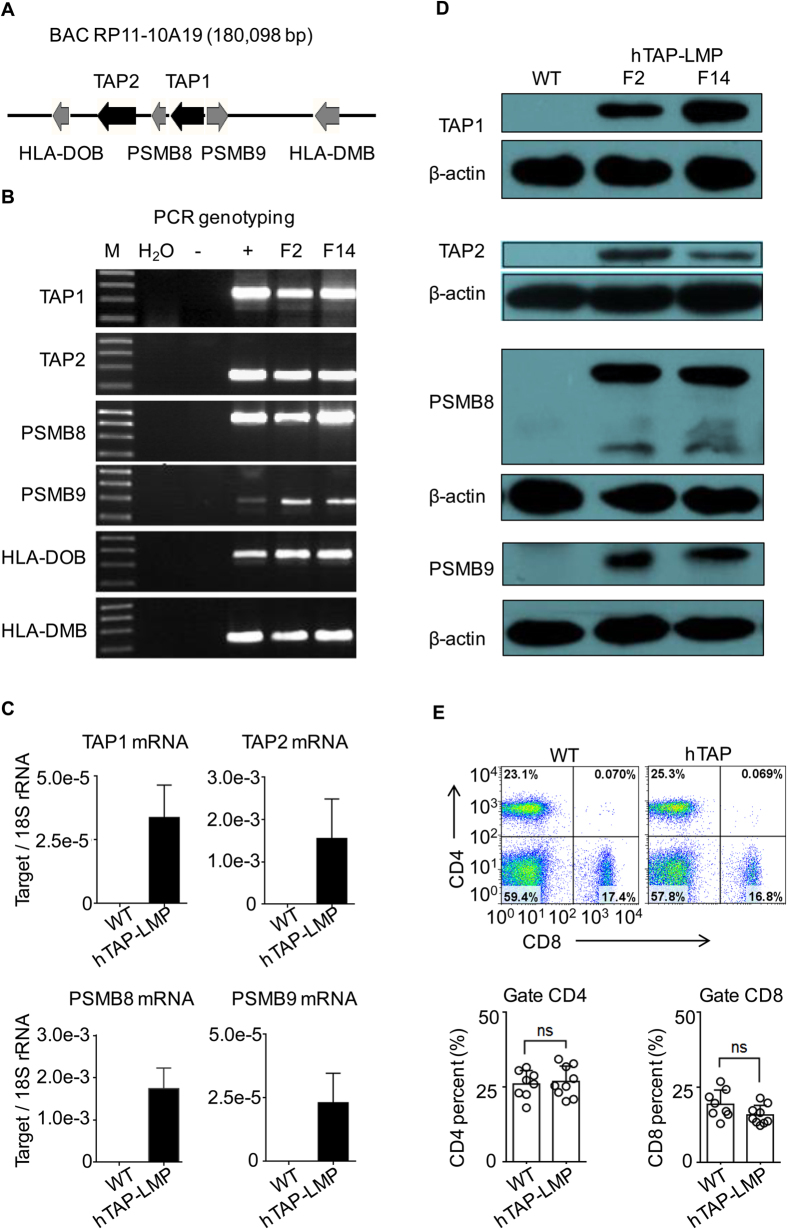
Generation of hTAP-LMP transgenic mice. (**A**) Schematic map of the BAC clone RP11-10A19. (**B**) PCR genotyping of the founder mice. F2 and F14 were the two positive founder mice. “−”, tail DNA samples of WT littermates; “+”, plasmid DNA of the BAC clone RP11-10A19. (**C**) Relative mRNA expression of human TAP1, TAP2, PSMB8, and PSMB9 in splenocytes from WT or hTAP-LMP mice as analyzed by qRT-PCR and normalized to 18S rRNA levels. (**D**) Expression of human TAP1 (65 kDa), TAP2 (75 kDa), PSMB8 (23/28 kDa), and PSMB9 (22 kDa) in WT or hTAP-LMP mice was determined by western blotting; β-actin (42 kDa) was used as an internal control. (**E**) FACS analysis for CD4 and CD8 expression of splenocytes from WT and hTAP-LMP mice (up), with the percentage of CD4^+^ T cells (below, left) and CD8^+^ T cells (below, right). PCR genotyping and western blotting were performed on individual mice, and the depicted results are representative of at least three independent experiments. Full-length PCR gels and Western blots are presented in Supplementary Figs S6 and S7, respectively. Results of the qRT-PCR and FACS analysis are representative of at least three independent experiments with n ≥ 3. Data represent the mean ± SD. ns, not significant.

Differences between human and mouse antigen processing and presentation machinery raise the possibility that HLA-I-restricted antigen presentation is not intact in HLA-I transgenic mice^8^. Thus, we generated a novel humanized TAP-LMP transgenic mouse by microinjecting pronuclei with a human BAC clone (RP11-10A19) encoding the intact hTAP-LMP gene cluster (Fig. 1A). Sequence alignment of BAC RP11-10A19 revealed that this BAC clone carries TAP1*0101 and TAP2*0201, both of which occur at high frequencies in different human populations^31^,^32^. By using PCR screening, two founder mice (F2 and F14) carrying all six human genes were established (Fig. 1B). mRNA and protein expression of human TAP1, TAP2, PSMB8, and PSMB9 in both founder mice were further confirmed by using real-time PCR and western blotting, respectively (Fig. 1C,D). Additionally, the human TAP1 mRNA expression level in the transgenic mice was approximately equal to that in human PBMC (Supplementary Fig. S1). Transgenic expression of this hTAP-LMP gene cluster had little effect on mouse T cell homeostasis, as indicated by the normal percentage of CD4^+^ and CD8^+^ T cells found in the spleens and thymus of hTAP-LMP transgenic mice (Fig. 1E and Supplementary Fig. S2). The transgenic mice of the F14 founder were kept and used in further experiments.

### Enhancement of HLA class I expression in HLA-I/hTAP-LMP transgenic mice

**Figure 2:**
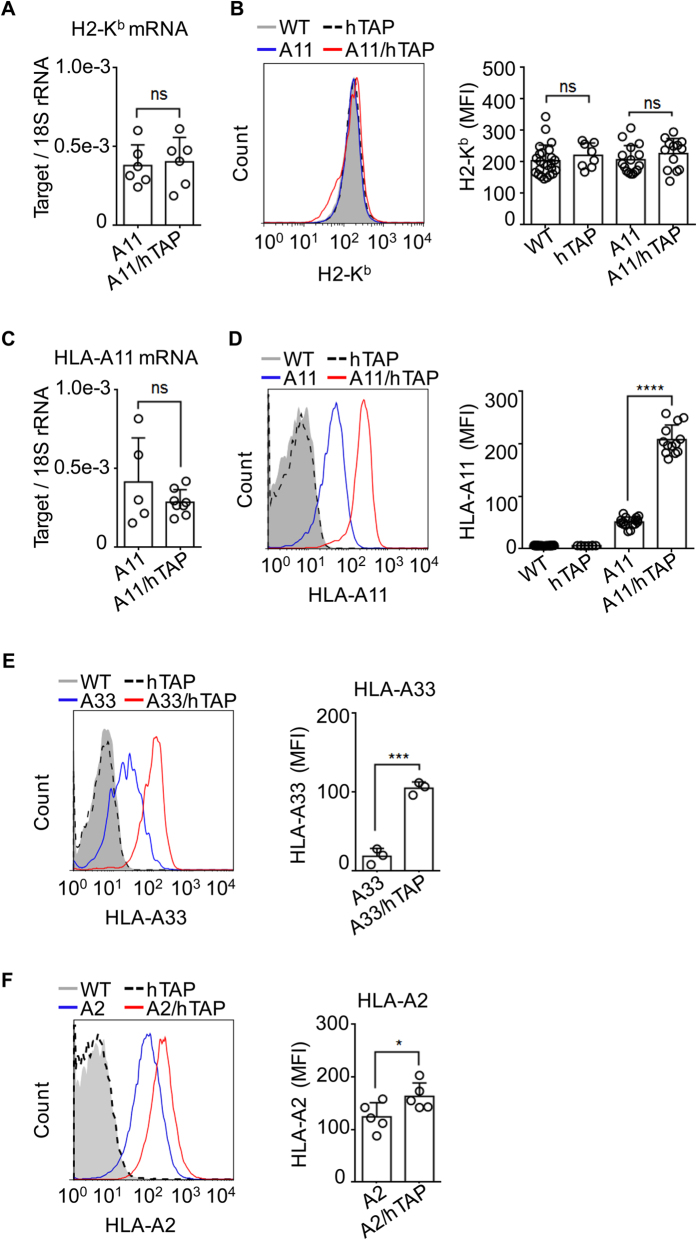
High expression levels of HLA-I molecules in HLA-I/hTAP-LMP double transgenic mice. Splenocytes from HLA-A11 and HLA-A11/hTAP-LMP mice were isolated and used for mRNA extraction to analyze H2-K^b^ or HLA-A11 mRNA expression by qRT-PCR. Surface expression of H2-K^b^ and HLA-AI was determined using flow cytometric analysis. (**A**) Relative expression of H2-K^b^ mRNA in splenocytes of HLA-A11 and HLA-A11/hTAP-LMP mice. (**B**) Representative histogram of H2-K^b^ expression and cumulative data for H2-K^b^ (MFI) in WT, hTAP-LMP, HLA-A11, and HLA-A11/hTAP-LMP mice. (**C**) Relative expression of HLA-A11 mRNA in splenocytes of HLA-A11 and HLA-A11/hTAP-LMP mice as analyzed by qRT-PCR. (**D**) Representative histogram of HLA-A11 expression in WT, hTAP-LMP, HLA-A11, and HLA-A11/hTAP-LMP mice (left) and cumulative data for HLA-A11 (MFI) in HLA-A11 and HLA-A11/hTAP mice (right). (**E**) Representative histogram of HLA-A33 expression in WT, hTAP-LMP, HLA-A33, and HLA-A33/hTAP-LMP mice (left) and cumulative data for HLA-A33 (MFI) in HLA-A33 and HLA-A33/hTAP mice (right). (**F**) Representative histogram of HLA-A2 expression in WT, hTAP-LMP, HLA-A2, and HLA-A2/hTAP-LMP mice (left) and cumulative data for HLA-A2 (MFI) in HLA-A2 and HLA-A2/hTAP mice (right). Each symbol represents data from one animal; MFI, mean fluorescence intensity. Data represent the mean ± SD. The Results are representative of at least three independent experiments with n ≥ 3. *p < 0.05, ***p < 0.001, and ****p < 0.0001, unpaired *t*-test. ns, not significant.

We next set out to test whether expression of the hTAP-LMP gene cluster promoted HLA-I-restricted antigen presentation. HLA-A3 supertypes, including A11 and A33, which prefer to bind peptides with small or aliphatic residues at position 2 and basic residues at the C-terminus (R or K)^27^, are more likely to rely on human TAP than other HLA supertypes. In addition, an HLA-A11 transgenic mouse that is a representative mouse model for the A3 supertype displays a defect in processing natural A11-restricted epitopes^5^,^29^. Therefore, hTAP-LMP mice were crossed with HLA-A11 transgenic mice to generate HLA-A11/hTAP-LMP double transgenic mice, and endogenous antigen presentation was evaluated by surface staining of HLA-A11. We found that the expression of surface mouse class I (H2-K^b^) protein and mRNA was not significantly affected by transgenic expression of hTAP-LMP (Fig. 2A,B). In contrast, surface HLA-A11 molecules but not their mRNA levels were strikingly increased in the HLA-A11/hTAP-LMP transgenic animals (Fig. 2C,D). Indeed, approximately four-fold higher HLA-A11 levels were found in the HLA-A11/hTAP-LMP animals than the control HLA-A11 mice (Fig. 2D). This result suggests that reconstitution of humanized TAP-LMP enabled more HLA-A11-matched peptides to be transported into the ER to stabilize HLA-A11 molecules and increase their restricted antigen display.

The promotion of HLA-I antigen presentation by hTAP-LMP was highly selective, as we also found dramatic enhancement of another member of the A3 subtype, HLA-A33 molecules, in the HLA-A33/hTAP-LMP double transgenic mice (Fig. 2E), whereas the surface presentation of HLA-A2 only showed a slight up-regulation in HLA-A2/hTAP-LMP mice (Fig. 2F). This is consistent with their different peptide binding preferences (*i.e.*, binding to peptides with positive charges at their C-termini). The HLA-A2 molecule also has its own peptide in the signal sequence that does not require cytosolic processing or TAP transport^33^,^34^ and, thus, is less affected by the transgenic hTAP-LMP genes.

### Increased CTL responses against HLA-A11-restricted epitopes in the HLA-A11/hTAP-LMP mice

**Figure 3:**
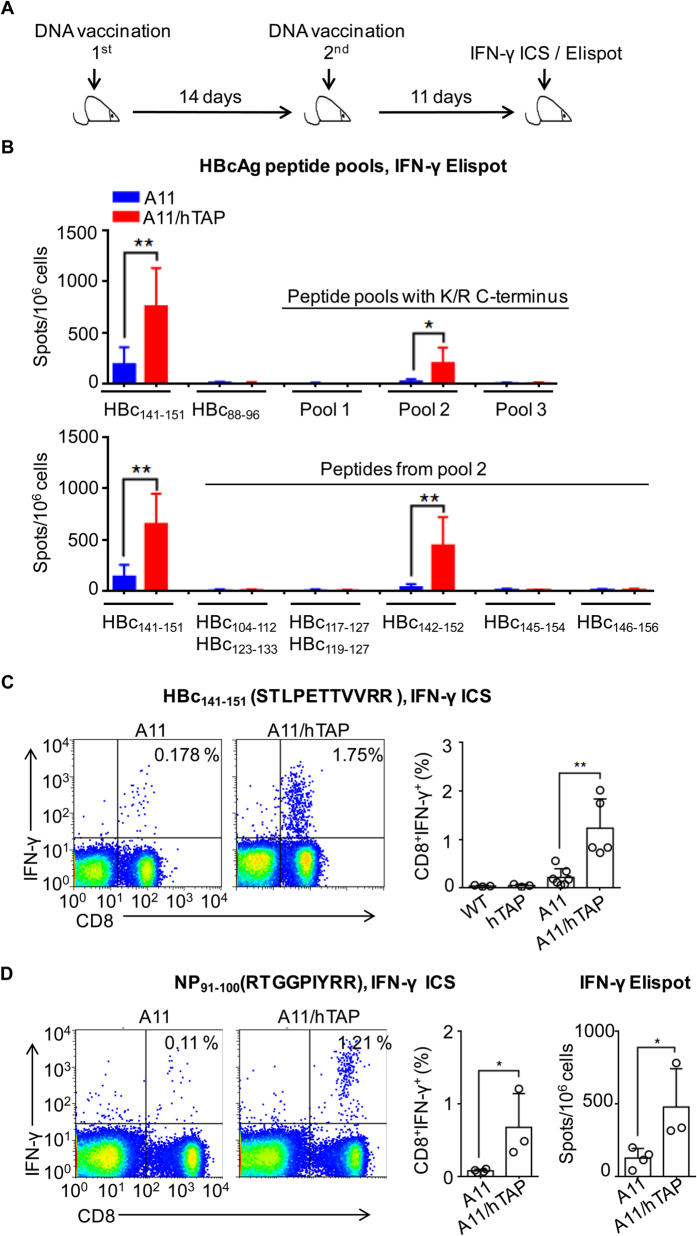
Increased CTL response against HLA-A11-restricted epitopes in the HLA-A11/hTAP-LMP mice. **(A)** Experimental outline for DNA vaccination and subsequent assessment of HLA-A11-restricted CTL responses. (**B**) HLA-A11/hTAP-LMP and control HLA-A11 mice were vaccinated twice with 100 μg of pcDNA3.1(+)/HBcAg, and then splenocytes were separated and analyzed for CTLs against HLA-A11-restricted epitopes using IFN-γ Elispot assays. Two published HLA-A11 restricted epitopes, HBc_141-151_ (STLPETTVVRR, 14) and HBc_88-96_ (YVNTNMGLK, 67), and 19 peptides with K/R C-termini that potentially elicit HLA-A11-restricted CTL responses were synthesized and used in the Elispot assays. Initially, the 19 peptides were divided into three pools for *ex vivo* stimulation (Table 1). Single or overlapping peptides from pool 2 were further used to screen for HLA-A11-restricted epitopes. **(C)** Splenocytes as in (**B**) were used to test for HBc_141–151_-specific CD8^+^ T cells by ICS. The left plot shows the representative FACS diagram, indicating the percentage of CD8^+^ IFN-γ^+^ cells in the total CD8^+^ T cells; the right chart depicts the cumulative data. **(D)** A minigene encoding a known HLA-A11-restricted peptide, NP_91–100_ (RTGGPIYRR), was used to vaccinate HLA-A11 and HLA-A11/hTAP-LMP mice. The NP_91–100_-specific CTL response was tested by IFN-γ ICS (left) and IFN-γ Elispot assays (right). The results are representative of at least three independent experiments with n ≥ 3. Data represent the mean ± SD. *p < 0.05 and **p < 0.01, unpaired *t*-test.

To test whether the introduction of the human TAP-LMP gene cluster would have an impact on HLA-A11-restricted CTL responses and to better establish a link between TAP affinity, HLA-I expression, and CTL responses, we utilized DNA vaccination to allow antigens to be processed and presented in the intracellular pathway. HLA-A11/hTAP-LMP mice were prime-boost immunized via intramuscular injection of plasmid pcDNA3.1(+)/HBcAg, which encodes the full-length hepatitis B virus core antigen (HBcAg) (Fig. 3A). HLA-A11-restricted CTLs were evaluated by Elispot assays and intracellular IFN-γ cytokine staining (ICS). Two known HLA-A11-restricted epitopes, HBc_141–151_ (STLPETTVVRR)^5^ with high affinity to human TAP (TAP score 0.67, IEDB Analysis Resource, http://tools.iedb.org/processing/) and HBc_88–96_ (YVNTNMGLK)^35^ with low TAP affinity (TAP score 0.15), as well as 19 peptides (Table 1) with K/R C-termini that potentially elicit HLA-A11-restricted CTL responses, were synthesized and used in the Elispot assays (Fig. 3B). Initially, the 19 peptides were divided into three pools for *ex vivo* stimulation (Table 1). Because mouse class I molecules prefer to bind peptides with hydrophobic C-termini, the studied peptides were unlikely to trigger mouse class I-restricted CTLs.

Consistent with a previous influenza virus infection model^5^, only weak HLA-A11-restricted CTL responses were detected by Elispot assays in the A11 transgenic mice following DNA vaccination (Fig. 3B above). However, much stronger CTL responses against HBc_141–151_ (but not HBc_88–96_) and pool 2 peptides were found in HLA-A11/hTAP-LMP mice (Fig. 3B above). Interestingly, further analysis revealed that the only peptide from pool 2 that was capable of stimulating the IFN-γ response was HBc_142–152_ (Fig. 3B below). The HBc_142–152_ peptide was derived from the same region as HBc_141–151_, with the differences being that it lacks a serine at its N-terminus and has an additional arginine at its C-terminus. This suggests that HLA-A11-restricted epitopes in the HBcAg DNA vaccination model dominantly reside between residues 141 and 152. Moreover, a much higher number of HBc_141–151_ epitope-specific CTLs detected via ICS was also found in HLA-A11/hTAP-LMP mice, highlighting the importance of humanized TAP-LMP in the HLA-A11-dependent CTL response (Fig. 3C).

Similarly, by DNA vaccination of another plasmid encoding a minigene that contains a known HLA-A11-restricted epitope, NP_91–100_ (RTGGPIYRR)^36^ with high TAP affinity (TAP score 0.62), a stronger NP_91–100_-specific CTL response was also found in HLA-A11/hTAP-LMP mice as analyzed by ICS (Fig. 3D, left and middle panel) and IFN-γ Elispot assays (Fig. 3D, right panel). We then detected the CTL responses to the HLA-A2 restricted epitope HBc_18–27_ (FLPSDFFPSV) with low TAP affinity (TAP score 0.07)^37^. There was no significant difference between HLA-A2/hTAP-LMP mice and HLA-A2 mice (Supplementary Fig. S3). Overall, our results indicated that the introduction of hTAP-LMP prompts better intracellular antigen presentation of HLA-A11 molecules and notably improved HLA-A11-restricted CTL responses to epitopes with high affinity for human TAP.

### Antigen presentation of HLA-A11-restricted epitopes in a long peptide vaccine elicited anti-viral immunity

**Figure 4:**
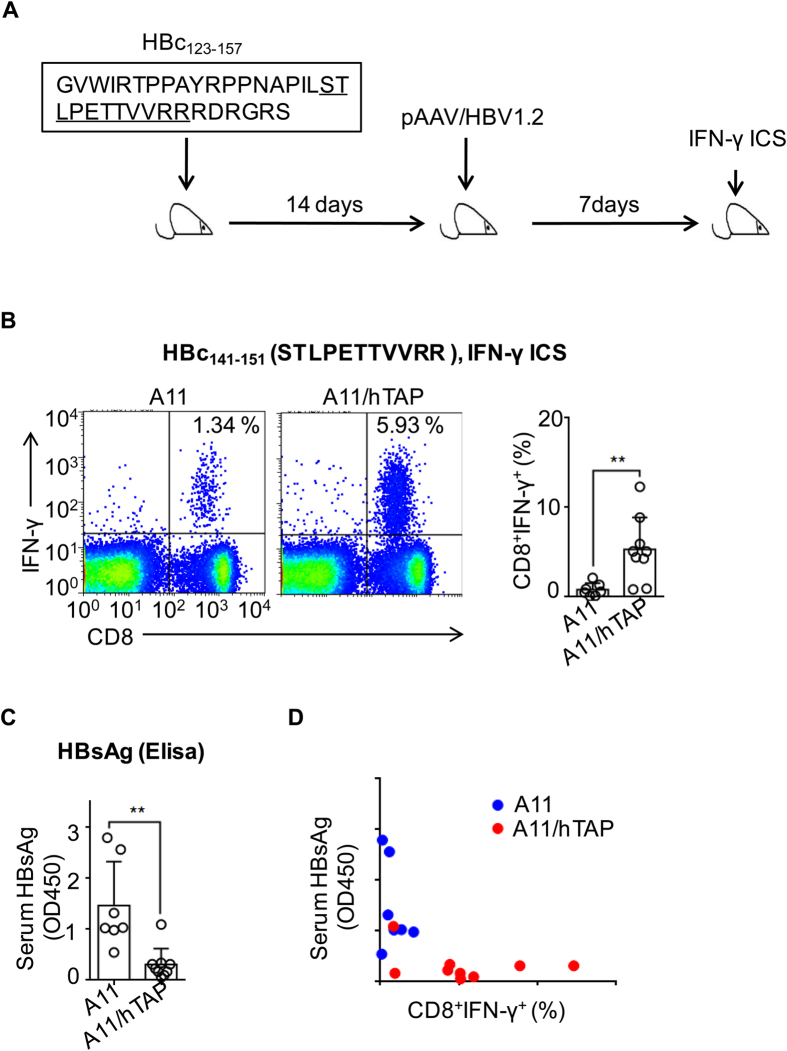
Long peptide vaccine HBc_123–157_ elicited strong anti-viral immunity in the HLA-A11/hTAP-LMP mice. **(A)** Experimental outline for long peptide vaccination. **(B)** HLA-A11 and HLA-A11/hTAP-LMP mice were subcutaneously (s.c.) injected with 100 μg of HBc_123–157_. Two weeks later, 10 μg of pAAV/HBV1.2 was hydrodynamically injected to mimic HBV infection. HBc_141–151_-specific CD8^+^ T cells were determined using IFN-γ ICS. The left plot shows the representative FACS diagram, indicating the percentage of CD8^+^ IFN-γ^+^ cells in the total CD8^+^ T cells; the right chart depicts the cumulative data. (**C**) Relative serum HBsAg levels of the mice in (**B**) were determined by using ELISAs at day 7 after HDI. (**D**) Correlation plot of relative serum HBsAg and the percent of HBc_141–151_-specific CD8^+^ T cells in HLA-A11 or HLA-A11/hTAP-LMP mice of (**B**,**C**). Each symbol represents data from one animal. Data represent the mean ± SD. **p < 0.01, unpaired *t*-test.

HBc_141–151_ is a CTL epitope shared by other class I alleles in the HLA-A3 supertype^5^,^38^. In addition, this epitope elicits a specific CTL response that is correlated with HBV clearance^39^,^40^. To explore the possibility of boosting the HBc_141–151_-specific CTL response as an intervention method to inhibit hepatitis B virus infection, we designed a long peptide vaccine containing residues 123–157 of HBcAg (HBc_123–157_, Fig. 4A), which encompassed the HBc_141–151_ CTL epitope. Unlike short peptides, long peptide vaccines (which are capable of forming tertiary structure to protect the peptides from exopeptidase degradation^41^) are predicted to be internalized and cross-presented by professional APCs. To test potential advantages of the long peptide HBc_123–157_, HLA-A11/hTAP-LMP mice and a control strain were subcutaneously immunized with the long peptide HBc_123–157_. Two weeks later, hydrodynamic injection (HDI) of pAAV/HBV1.2 was used to mimic HBV infection *in vivo*^42^ (Fig. 4A). The plasmid pAAV/HBV1.2 contains a replication-complement HBV DNA sequence that can mimic HBV infection after liver-targeting HDI into mice. The mouse models will help to further explore new treatment of HBV infections^42^.

The HBc_141–151_-specific CTL response was determined by ICS, and serum HBV surface protein (HBsAg) levels were evaluated by ELISAs 7 days later. After HDI, much higher numbers of HBc_141–151_-specific IFN-γ secreting CD8^+^ T cells were found in splenocytes from HLA-A11/hTAP-LMP mice (Fig. 4B), which was inversely correlated with the serum HBsAg levels (Fig. 4C,D). IFN-γ secreting CD8^+^ T cells were HLA-A11 Tetramer^+^ ki67^+^, and also expressed cytotoxicity marker CD107a, indicated they are bona fide CTL (Supplementary Fig. S4).These results thus demonstrated that the long peptide vaccination boosted a functional HBc_141–151_-specific CTL response that promoted HBV clearance *in vivo*. Our results emphasize that the HLA-I/hTAP-LMP mouse model is an advanced experimental model for assessing HLA-I-restricted CTLs in response to naturally processed epitopes, which can be used as a new vaccine development platform in the future.

## Discussion

In the last two decades, HLA-I transgenic mice have proven to be a unique *in vivo* model to study human class I-restricted CTL responses in various infectious diseases^6^,^29^, as well as cancer immunotherapy^43^. However, the murine antigen processing and presentation machinery is not capable of completely mimicking human HLA-I-restricted antigen presentation. In this study, a novel BAC transgenic mouse carrying the human TAP1, TAP2, PSMB8, and PSMB9 genes (hTAP-LMP mice) was generated, and we found that reconstitution of the hTAP-LMP gene cluster notably improved human HLA-I antigen presentation and restricted CTL responses. This effect was especially evident in the A3 supertype. Our data support the notion that particular HLA-I molecules co-evolved with TAP-LMP for efficient peptide processing and presentation. This research also highlights the potential for HLA-I/hTAP-LMP mice as an improved experimental model for studying antigen presentation of HLA-I molecules and their related immune responses.

HBV infection is the most common liver disease in the world. More than 350 million individuals are infected with HBV, and the estimated number in China alone is close to 100 million^44^,^45^. HLA A*1101, a member of the A3 supertype, is the major HLA-I allele in chronic hepatitis B patients from China^46^. Thus, identification of HLA-A*11:01-restricted HBV epitopes that can boost protective CTL responses are important for the treatment of chronic HBV infection. Here, using our novel HLA-A11/hTAP-LMP mice, we demonstrated that a long peptide vaccine containing residues 123–157 of HBcAg (HBc_123–157_) could be efficiently presented to APCs and elicit protective HBc_141–151_-specific CTL responses. Thus, HBc_123–157_ could have important therapeutic potential in preventing HBV infection. This result also suggests that the long peptide was more efficiently cross-presented in HLA-A11/hTAP-LMP mice, which is consistent with previous research showing that cross antigen presentation of long peptides is dependent on proteasome and TAP function^47^. However, a recent study by Ma *et al*.^48^ found that cross-presentation of long peptides requires a vacuolar pathway that depends on newly synthesized MHC class I molecules but not the proteasome or TAP molecules^48^. Thus, further studies are needed to clarify whether enhanced cross antigen presentation of long peptides in HLA-A11/hTAP-LMP mice is associated with transgenic human TAP and LMP molecules.

Because the TAP-LMP gene cluster plays important roles in HLA-I antigen presentation^30^, future studies on the effect of hTAP-LMP on HLA-I-related human diseases are particularly interesting. HLA-A33 molecules are related to susceptibility to persistent infection by HBV^49^,^50^ and Enterovirus 71 infection^51^. It will be of great interest to use both HLA-A33/hTAP-LMP and HLA-A33 transgenic mice to study the contributions of HLA-A33 molecules to virus infection.

Though the TAP and LMP molecules are tightly linked as a gene cluster in HLA-I/hTAP-LMP mice, it is interesting to clarify their different contributions to antigen presentation of HLA-I molecules. One of the most obvious effects from the transgenes is that the HLA-A11 expression levels were dramatically elevated in HLA-A11/hTAP-LMP mice. To determine whether this effect is due to the human TAP transgene, splenocytes of HLA-A11/hTAP-LMP mice were infected with a retrovirus expressing a HSV-2 protein ICP47-2 which was reported to specifically inhibit human TAP^52^. Interestingly, HLA-A11 expression was reduced robustly and sensitively in splenocytes that were successfully infected by ICP47-2 exressing virus, while the H2-K^b^ expression displayed a less sensitive manner (Supplementary Fig. S5). The reduction of H2-K^b^ expression was expected because ICP47-2 also inhibits murine TAP transport^52^. Importantly, in a previous study, inhibition of human TAP by ICP47 molecules cound downregulate HLA class I in human cells^53^. Thus, it is quite possible that transgenic human TAP molecules make a substantial contribution to the elevation of HLA-A11 molecules and the induction of strong CTL responses. Regardless, whether the human LMP molecules have a similar effect is uncertain and will be tested in further studies.

In conclusion, we demonstrated that HLA-I/hTAP-LMP transgenic mice are an efficient *in vivo* model for studying HLA-I antigen presentation and CTL responses. We expect that these mice will be useful tools for future vaccine development and cancer immunotherapy.

## Methods

### DNA Constructs and peptides

The bacterial artificial chromosome (BAC) clone RP11-10A19 was purchased from CHORI (Oakland, CA, USA), pAAV/HBV1.2 was kindly provided by Pei-Jer Chen^42^, pcDNA3.1(+)/HBc (type D) was kindly provided by Songdong Meng, and pcDNA3.1(+)/minigene encoding the peptide NP_91–100_ was constructed in the lab. The peptides HBc_141–151_ (STLPETTVVRR), NP_91–100_ (KTGGPIYRR), HBc_128–140_ (TPPAYRPPNAPIL), HBc_123–157_ (GVWIRTPPAYRPPNAPILSTLPETTVVRRRDRGRS), and the 19 peptides with K/R C-termini were synthesized by the Beijing Xuheyuan Company with a purity of >90%.

### Mice

BAC RP11-10A19 was microinjected into pronuclei of fertilized B6 × DBAF1 mouse oocytes to generate human TAP transgenic mice (hTAP-LMP mice). The hTAP-LMP mice were further back crossed at least seven generations to C57BL/6. HLA-A2 transgenic mice in the C57BL/6 background were kindly provided by Songdong Meng’s lab. HLA-A11 transgenic mice were purchased from Taconic (Model# 9660). HLA-A33 transgenic mice were generated in our lab by microinjection of the Rosa26 BAC (RP24-85L15 CHORI, Oakland, CA, USA) inserted with a HLA-A*3303/K^b^ fused gene into pronuclei of fertilized B6 × DBAF1 mouse oocytes, and the mice were back crossed at least seven generations to C57BL/6. HLA-A2/hTAP-LMP, HLA-A11/hTAP-LMP, and HLA-A33/hTAP-LMP mice were obtained by crossing HLA-A2, HLA-A11, and HLA-A33 mice with hTAP-LMP mice, respectively. All transgenic mice were maintained as heterozygotes. All mice were housed under specific pathogen-free conditions at the Institute of Microbiology, Chinese Academy of Sciences in accordance with the guidelines for care and use of laboratory animals established by the Beijing Association for Laboratory Animal Science. All mouse experiments were performed in accordance with the “Regulation of the Institute of Microbiology, Chinese Academy of Sciences of Research Ethics Committee”. The protocol was approved by the Research Ethics Committee of the Institute of Microbiology, Chinese Academy of Sciences (permit number PZIMCAS2012003).

### PCR and western blotting

The primers for PCR genotyping of hTAP-LMP mice were: TAP1-F: 5′-GCCTCTTATCGTGTGCTTCT-3′, TAP1-R: 5′-CCATCTCCACCCAAGGTC-3′; TAP2-F: 5′-CGCCCACAGTGTTAGGT-3′, TAP2-R: 5′-GAAGAGGCGTTTGGAATAG-3′; PSMB8-F: 5′-AGTACGTCAGGTATTTAGC-3′, PSMB8-R: 5′-GAGGGAGTAGGAGTATATG-3′; PSMB9-F: 5′-CACTTAATGTCTCAGTGGGA-3′, PSMB9-R: 5′-TATTTCTCTTCCTGTTCCTC-3′; HLA-DOB-F: 5′-TAAACGTGTCTGGTATGTC-3′, HLA-DOB-R: 5′-AGTTAAAGATGAATCTGACC-3′; HLA-DMB-F: 5′-CAAAGGAACATCCAGATGAAG-3′, and HLA-DMB-R: 5′-TCCCCCATACTCCCTGAAG-3′. The PCR products were 319, 234, 438, 289, 347, and 228 bp for human TAP1, TAP2, PSMB8, PSMB9, HLA-DOB, and HLA-DMB, respectively.

Lysates of splenocytes from hTAP-LMP mice were used to detect human TAP1, TAP2, PSMB8, and PSMB9 expression by western blotting. An anti-human TAP1 mAb (clone 148.3, Cat MABF125, Merck Millipore), anti-human TAP2 mAb (clone TAP2.17, MBL), anti-human PSMB8 mAb (clone 1A5, CST), and anti-human PSMB9 mAb (clone 792520, R&D) were used as primary antibodies, and anti-mouse IgG/HRP (ZDR-5307, ZSGB-BIO, Beijing) was used as the secondary antibody. The mouse anti-β-actin antibody (DKM9001, Tianjin Sungene Biotech) was used to detect β-actin as an internal reference.

### Immunization and HDI

For intramuscular immunization, 100 μg of DNA vaccine (pcDNA3.1(+)/HBcAg or pcDNA3.1(+)/minigene) dissolved in 100 μL of PBS was injected into the tibialis anterior muscle (50 μL per leg), followed with electroporation at the injection site^42^. The injection was performed twice within a 2-week interval. Eleven days after the last injection, the mice were sacrificed, and splenocytes were used to analyze peptide-specific CTLs by IFN-γ Elispot or IFN-γ ICS. Subcutaneous immunization of peptides was performed as previously described^5^ with some modifications. Briefly, HBc_123–157_ (100 μg/mouse) and the helper IA^b^-restricted epitope HBc_128–140_ (100 μg/mouse) in PBS/5% DMSO were emulsified with IFA and subcutaneously injected (s.c.) into the base of the tail. Two weeks after vaccination, the mice were hydrodynamically injected with pAAV/HBV1.2 plasmids (10 μg/mouse) in PBS with a volume (mL) equivalent to 8% of the mouse body weight (g), as previously described^42^. The mice were sacrificed 7 days later, and splenocytes were used to analyze CTL responses by IFN- γ ICS. The serum from day 7 after HDI was used to analyze HBsAg levels by ELISA (SHANGHAI KEHUA BIO-ENGINEERING CO., LTD).

### Flow cytometry

The antibodies used for surface staining were anti-HLA-A2 FITC (clone BB7.2, Biolegend), anti-HLA ABC FITC (clone B9.12.1, Beckman), anti-mouse H2-K^b^ PE (clone AF6-88.5, Biolegend), anti-mouse CD4 APC (clone GK1.5, Biolegend), anti-mouse CD8 FITC (PE) (clone 53–6.7, Biolegend), and anti-mouse/human B220 PerCP-Cy5.5 (clone RA3-6B2, eBioscience). All surface staining was performed in the dark at 4 °C for 20 min. Mouse IFN-γ staining was performed in the dark at 4 °C for 30 min using anti-mouse IFN-γ APC (clone XMG1.2, eBioscience). After washing, cells were analyzed using a FACS Calibur (BD).

### ICS & Elispot

IFN-γ ICS was performed as described^54^. Briefly, splenocytes were resuspended in RPMI 1640 medium containing 10% FBS, and 10^6^ splenocytes per well were incubated in 96-well round-bottom plates (Costar) with the designated concentration of peptides in the presence of monensin (Biolegend) at 37 °C and 5% CO_2_ for 5 h. Subsequently, cells were stained for surface markers at 4 °C for 30 min, washed twice, fixed with 4% fixation buffer PFA at room temperature for 8 min, permeabilized with Perm buffer (0.1% Saponin in FACS buffer), and stained for intracellular IFN-γ using anti-mouse IFN-γ APC. After washing twice with Perm buffer, cells were suspended in FACS buffer and analyzed using a FACS Calibur (BD). When co-staining with mouse CD107a, 2 μL of FITC anti-mouse CD107a (clone 1D4B, Cat 121606, Biolegend) was added at the beginning of culturing.

Biotinylated HLA-A*11:01-STLPETTVVRR monomers were kindly provided by the NIH Tetramer Core Facility. Tetramer-HLA-A11-HBc_141–151_ was synthesized from monomers and APC Streptavidin (Cat 405207, Biolegend). Tetramer staining was performed in the dark at 4 °C for 30 min together with surface antibodies.

Mouse IFN-γ Elispot assays were performed according to the manufacturer‘s instructions (BD). Briefly, 4 × 10^5^ or 5 × 10^5^ splenocytes per well were incubated with the designated concentration of peptides at 37 °C and 5% CO_2_ for 20 h. The plates were then developed according to the manual and analyzed with an ImmunoSpot S5 Versa Analyzer (CTL). Results of spot-forming cells per well were transformed into spots/10^6^ cells.

### Isolation of PBMCs from whole blood

Human Peripheral blood (5 mL each) was obtained after signed, informed consent from two adult normal donors using a protocol approved by the Research Ethics Committee of Institute of Microbiology, Chinese Academy of Sciences. All methods involving human subjects or donors were performed in accordance with the “Regulation of the Institute of Microbiology, Chinese Academy of Sciences of Research Ethics Committee”. Peripheral blood (~0.3 mL each) from three hTAP-LMP mice were collected from tail vein in presence of EDTA as anticoagulant. Both human and mouse peripheral blood mononuclear cells (PBMCs) were obtained by Ficoll centrifugation (Tian Jin Hao Yang BIOLOGICAL Manufacture. CO., LTD, LTS1077 for human and LTS1092 for mouse). Briefly, the blood was diluted with PBS (1:1 for human and 1:10 for mouse), and then carefully layered over the Ficoll medium (15 mL for human and 3 mL for mouse) in Falcon tube. The tube was then centrifuged at 500 g at room temperature for 20 min, no break. The PBMCs layer was then carefully transferred to a new Falcon tube and washed twice by PBS. The cells were counted and used for mRNA extraction (1*10^6^ cells/sample).

### RNA preparation and real-time PCR

Total mRNA from 5 × 10^6^ splenocytes/mouse was extracted using Trizol reagent (Invitrogen). A 2.5-μg aliquot of total mRNA per mouse was reverse-transcribed into cDNA (SuperScript III, Invitrogen). Specified mRNA expression was then analyzed by real-time PCR using a LightCycler480 System (Roche Applied Science), with a duplicate reaction volume of 10 μL for each well containing 5 μL of Mix 1, 2.5 μL of Mix 2 (Lightcycler480 Probe Master Mix kit, Roche 04707494001), 0.2 μL of probe (Taqman), 0.8 μL of primers, 0.5 μL of ddH2O, and 1 μL of 10-fold diluted cDNA. The reaction program was 95 °C for 10 min, followed by 45 cycles of 95 °C for 10 s, 60 °C for 30 s, and 72 °C for 1 s. The mRNA levels of different genes were analyzed by normalization against levels of 18S rRNA. The primers (and probes) used were: 5′-TCTCGCTGTTCCTGGTCCTGG-3′ and 5′-GTATCGGCTGAGCCATCTTGT-3′ for human TAP1 (probe #55), 5′-GGATGAAAAGCCCATCTCAC-3′ and 5′-GCTCCTGCCCAACTGAAAC-3′ for human TAP2 (probe #76), 5′-AAATGGAGAACGTATTTCA-3′ and 5′-CACGTAGTAGAGTCCAGGA-3′ for human PSMB8 (probe #5), 5′-ACCAACCGGGGACTTACC-3′ and 5′-ACCGCCTCGCCTGCAGACACT-3′ for human PSMB9 (probe #7), 5′-GTGATCTCTGGCTGTGAAGT-3′ and 5′-CTCCCACTTGTGTTTGGTGA-3′ for H2-K^b^ (probe #70), 5′-TAATGTATGGCTGCGACGTG-3′ and 5′-TCAGGGCGATGTAATCCTTG-3′ for HLA-A11 (probe #69), and 5′-GCAATTATTCCCCATGAACG-3′ and 5′-GGGACTTAATCAACGCAAGC-3′ for 18S rRNA (probe #48).

### Retrovirus infection

The ICP47-2 gene (GeneID:1487353, 261 bp) was synthesized by the Sangon Biotech Company (Shanghai) and inserted into the MSCV-IRES-NGFR (Addgene 27489) to construct MSCV-ICP47-NGFR. Virus production and infection was performed as described^55^ with some modifications. Briefly, 1.5 × 10^6^ of plat-E cells (producer cells) in a 6-well plate were co-transfected with 3 μg of MSCV-IRES-NGFR or MSCV-ICP47-NGFR and 1 μg of pCL-Eco plasmid using the Lipofectamine 2000 reagent (Cat 11668-019, Invitrogen). Viral supernatants were harvested 48 hours later and used to infect splenocytes activated by coated anti-CD3/anti-CD28 antibodies for 48 hours. Forty hours after virus infection, the splenocytes were collected and stained for HLA-A11 and NGFR expression level with anti-HLA ABC FITC antibody (clone B9.12.1, Beckman) and anti-human NGFR APC (clone ME20.4, Biolegend). Cells successfully infected by MSCV-ICP47-NGFR or MSCV-IRES-NGFR virus will be detected as NGFR positive.

### Statistical analyses

FACS data were collected and processed using FACS analysis software (FlowJo). Statistical analyses were performed using GraphPad Prism v6. The *p* values were determined using unpaired *t* tests; p < 0.05 was considered significant.

## Additional Information

**How to cite this article**: Huang, M. *et al*. Improved Transgenic Mouse Model for Studying HLA Class I Antigen Presentation. *Sci. Rep.* **6**, 33612; doi: 10.1038/srep33612 (2016).

## Supplementary Material

Supplementary Information

## Figures and Tables

**Table 1:** HBcAg peptides with K/R C terminus.

Position	Sequence	Pool
HBc18–28	FLPSDFFPSVR	Pool 1
HBc29–39	DLLDTASALYR	Pool 1
HBc47–56	HCSPHHTALR	Pool 1
HBc72–82	VGNNLEDPASR	Pool 1
HBc87–96	NYVNTNMGLK	Pool 1
HBc88–98	YVNTNMGLKIR	Pool 1
HBc90–98	NVNMGLKIR	Pool 1
HBc104–112	HISCLTFGR	Pool 2
HBc117–127	EYLVSFGVWIR	Pool 2
HBc119–127	LVSFGVWIR	Pool 2
HBc123–133	GVWIRTPPAYR	Pool 2
HBc142–152	TLPETTVVRRR	Pool 2
HBc145–154	ETTVVRRRDR	Pool 2
HBc146–156	TTVVRRRDRGR	Pool 2
HBc154–161	RGRSPRRR	Pool 3
HBc156–166	RSPRRRTPSPR	Pool 3
HBc161–168	RTPSPRRR	Pool 3
HBc169–177	RSQSPRRRR	Pool 3
HBc171–181	QSPRRRRSQSR	Pool 3
