# Supplementary material for: Improved Transgenic Mouse Model for Studying HLA Class I Antigen Presentation
Source: Sci Rep. 2016 Sep 16;6:33612. doi: 10.1038/srep33612 (PMC5025652; doi:10.1038/srep33612)
Supplement: Supplementary Information [file srep33612-s1.doc]

**Improved Transgenic Mouse Model for Studying HLA Class I Antigen Presentation**

Man Huang^1,2^, Wei Zhang^1^, Jie Guo^1^, Xundong Wei^1,2^, Krung Phiwpan^1,3^, Jianhua Zhang^1,*^, Xuyu Zhou^1,*^

^1^CAS Key Laboratory of Pathogenic Microbiology and Immunology, Institute of Microbiology, Chinese Academy of Sciences (CAS), Beijing, 100101, China; ^2^University of Chinese Academy of Sciences, Beijing, China; ^3^University of Phayao 19 Moo 2 Maeka, Muang Phayao district, Phayao 56000 Thailand

^*^Correspondence: Xuyu Zhou, Institute of Microbiology, Chinese Academy of Sciences F108, 1st Beichen West Road, Chaoyang, Beijing 100101, P. R. China; Email: [zhouxy@im.ac.cn](mailto:zhouxy@im.ac.cn); Tel: 86-10-64806075, Fax: 86-10-64807470. Jianhua Zhang, Institute of Microbiology, Chinese Academy of Sciences F110, 1st Beichen West Road, Chaoyang, Beijing 100101, P. R. China; Email: zhangjh@im.ac.cn; Tel: 86-10-64806070.

**
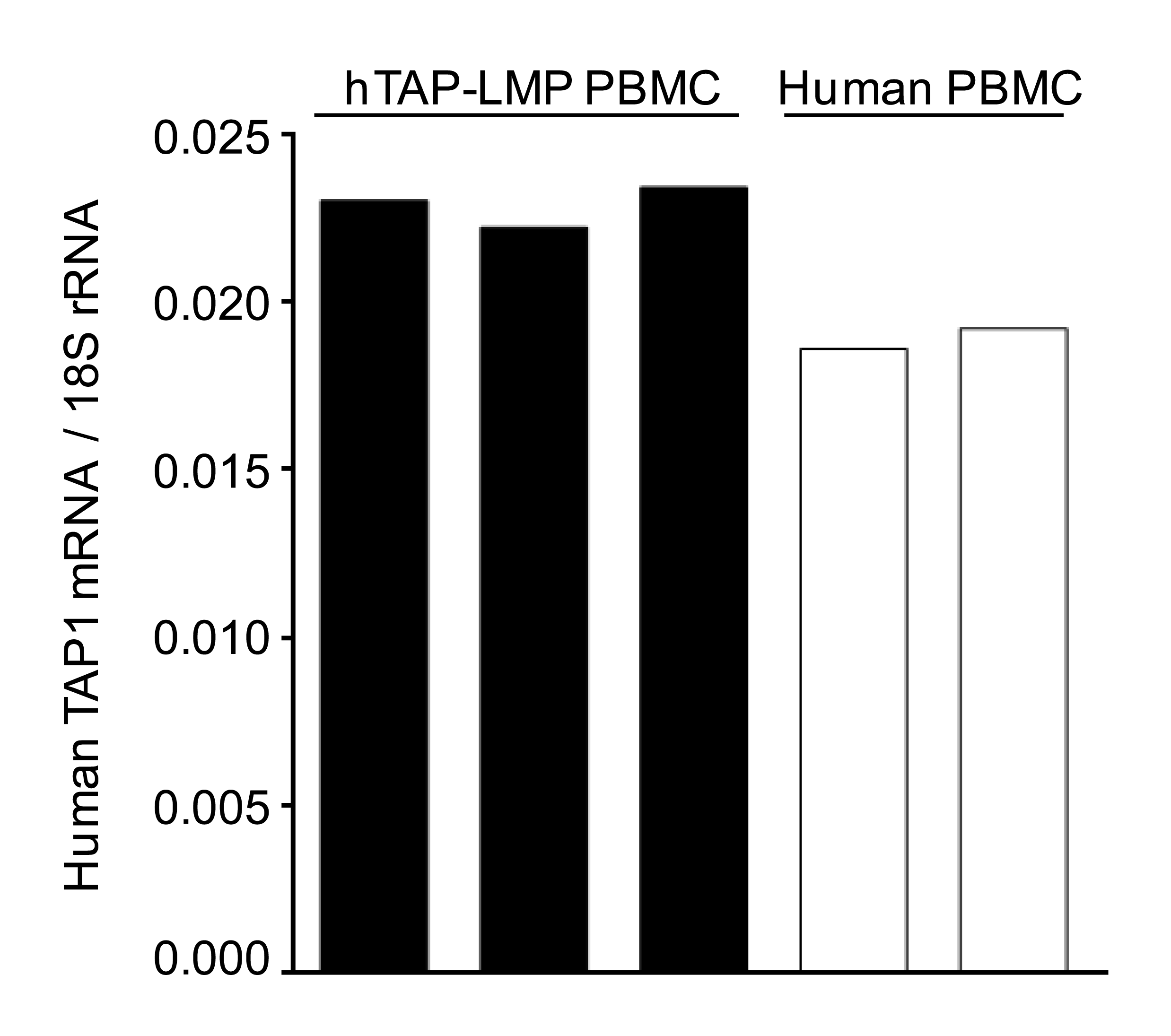
**

**Supplementary Figure 1.** **The hTAP-LMP transgenic mice express a approximately equal levels of human TAP1 mRNA to human.** mRNA expression level of human TAP1 in hTAP-LMP mice (PBMC, n=3, black) as well as human PBMC samples (n=2, empty) was analyzed by qRT-PCR and normalized to 18S rRNA levels.

**
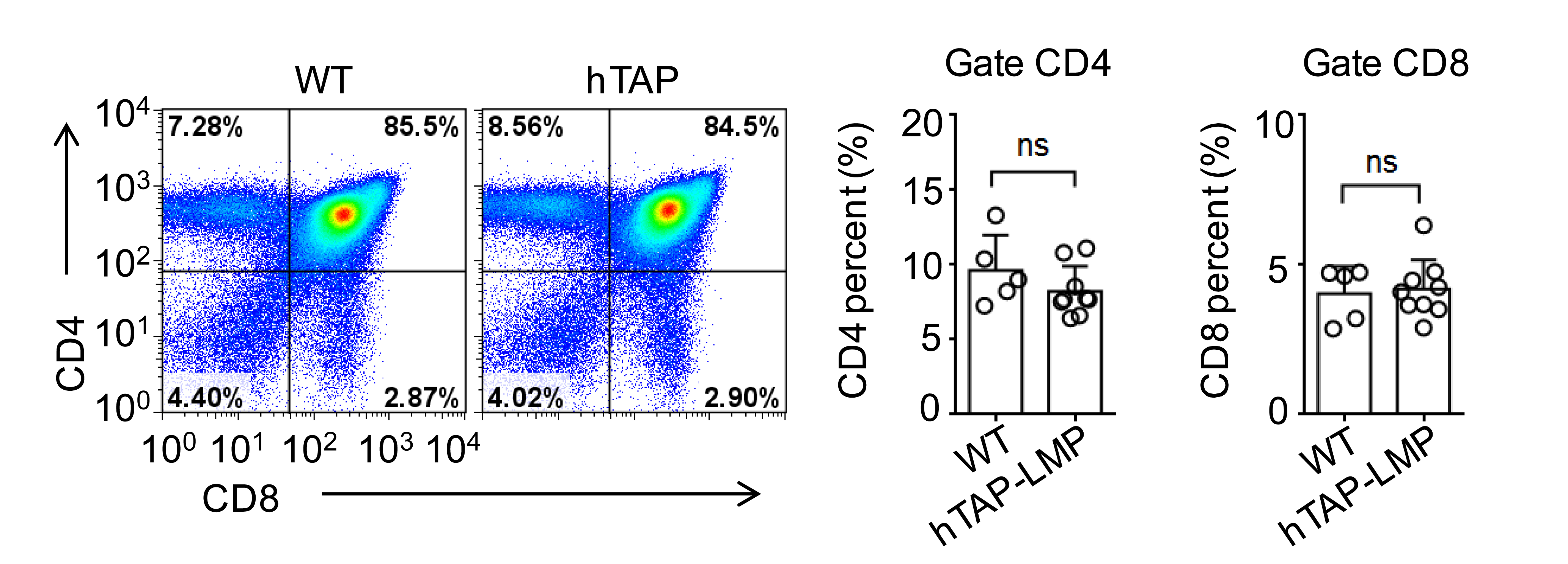
**

**Supplementary Figure 2**. **hTAP-LMP mice display normal numbers of CD4^+^ and CD8^+^ T cells in thymus.** CD4 and CD8 expression of thymocytes from WT (n=5) and hTAP-LMP (n=9) mice (left), with percentage of CD4^+^ T cells (middle) and CD8^+^ T cells (right).

**
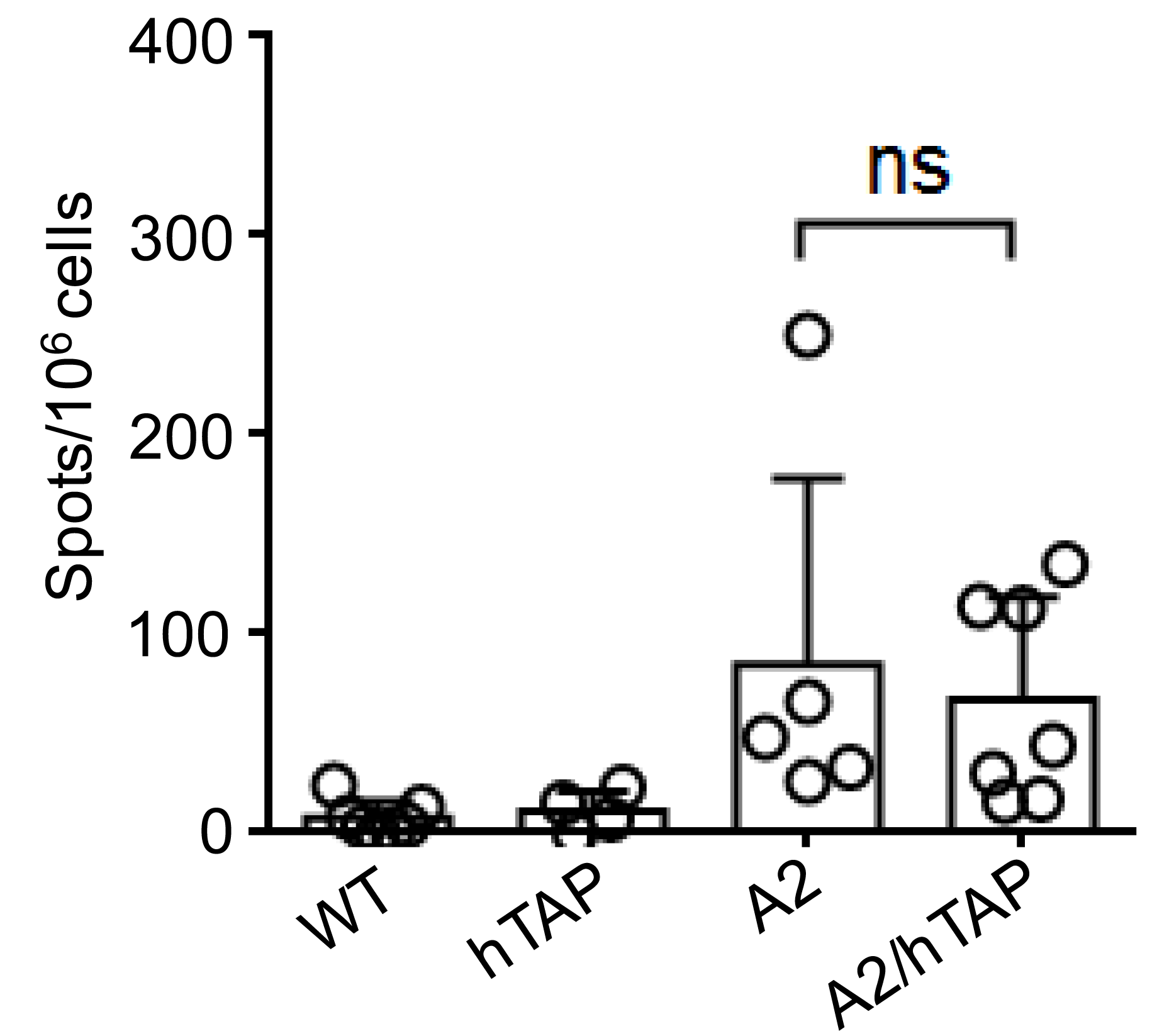
**

**Supplementary Figure 3**. **HLA-A2/hTAP-LMP and HLA-A2 mice display comparable CTL responses to HBc_18-27_.** After immunization of pcDNA3.1(+)/HBcAg (D type), the mice were sacrificed and splenocytes were harvested for analyzing HBc_18-27_ (FLPSDFFPSV) specific CTLs by IFN-γ Elispot. “ WT”, “hTAP”, “A2”, and “A2/hTAP” represent litermates of wild type (n=7), hTAP-LMP (n=4), HLA-A2 (n=5) and HLA-A2/hTAP-LMP (n=7) mice, respectively.


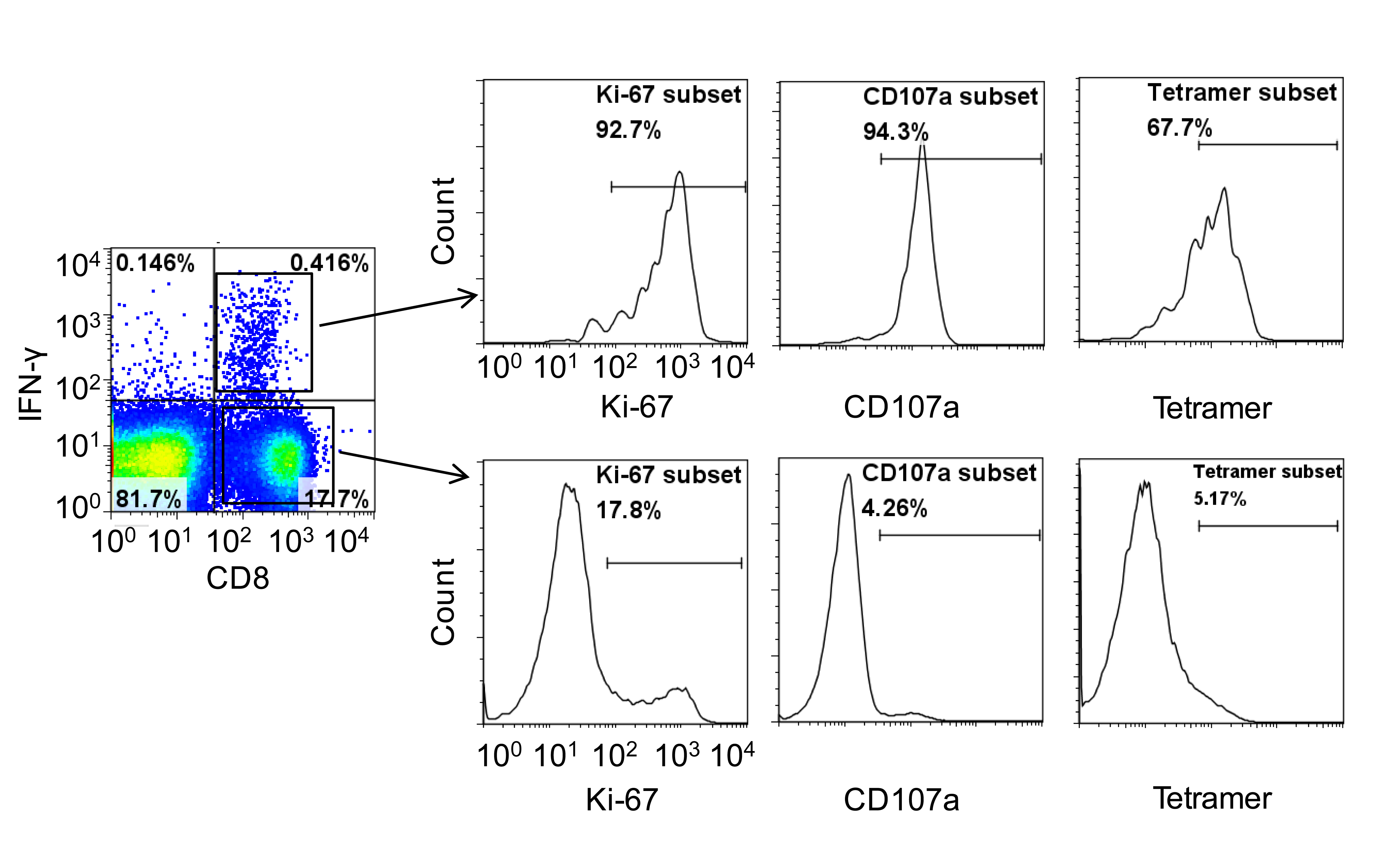


**Supplementary Figure 4.** **HBc_141-151_ specific CD8^+^IFN-γ^+^T cells display signatures of CTL proliferation and cytotoxicity.** After stimulation, CD8^+^IFN-γ^+^ T cells and CD8^+^ IFN^-^γ^-^ T cells from spleen were gated and analyzed for levels of Ki-67, CD107a and tetramer bindings. For tetramer-HLA-A11-HBc_141-151_ staining, lymphocytes from the liver were used.

**
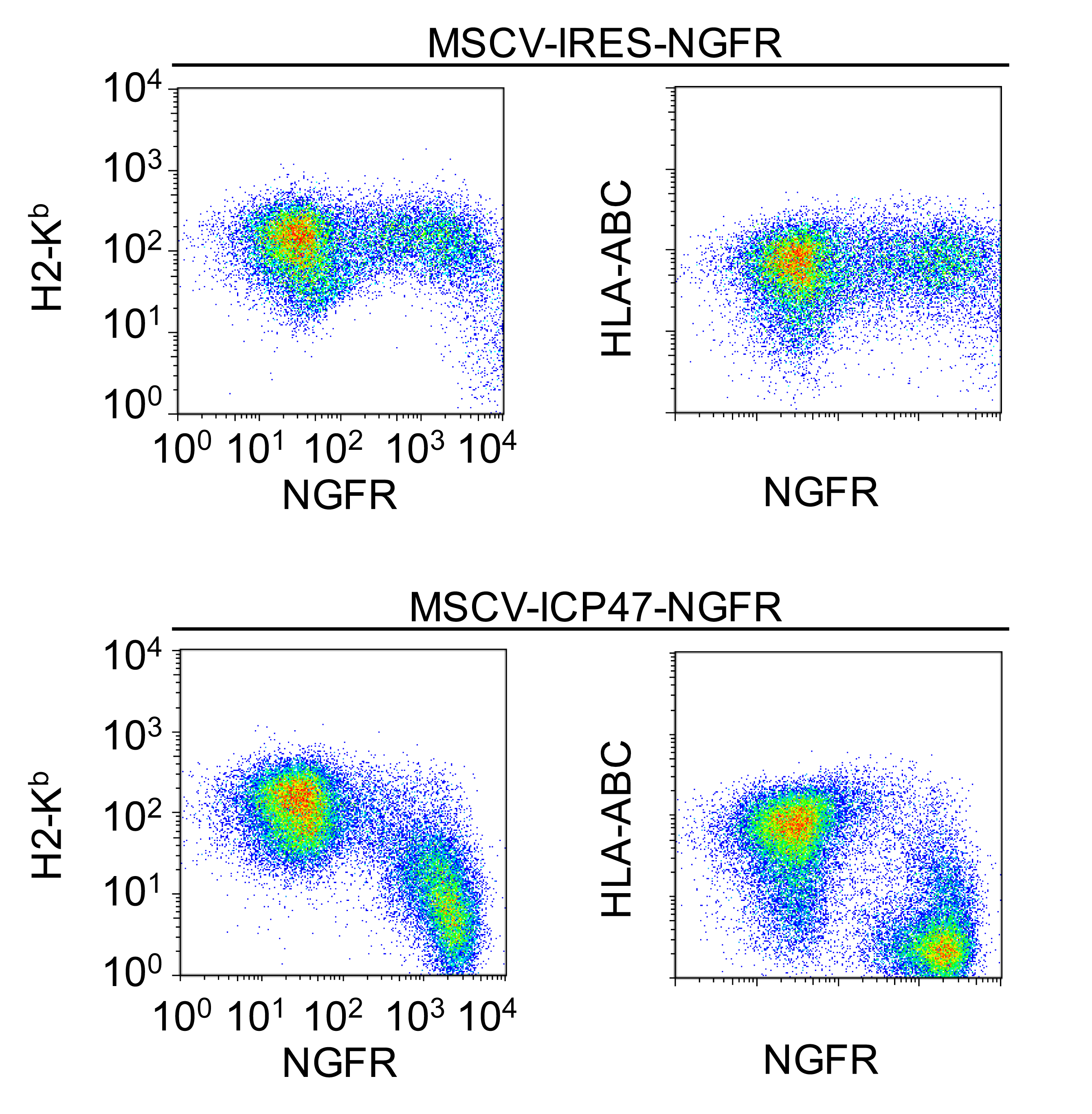
**

**Supplementary Figure 5: HLA-A11 expression level in HLA-A11/hTAP-LMP mice was dramatically reduced by inhibition of human TAP molecules.** Representative staining of H2-K^b^ and HLA-A11 expression on MSCV-IRES-NGFR (up) or MSCV-ICP47-NGFR (below) infecting cells.


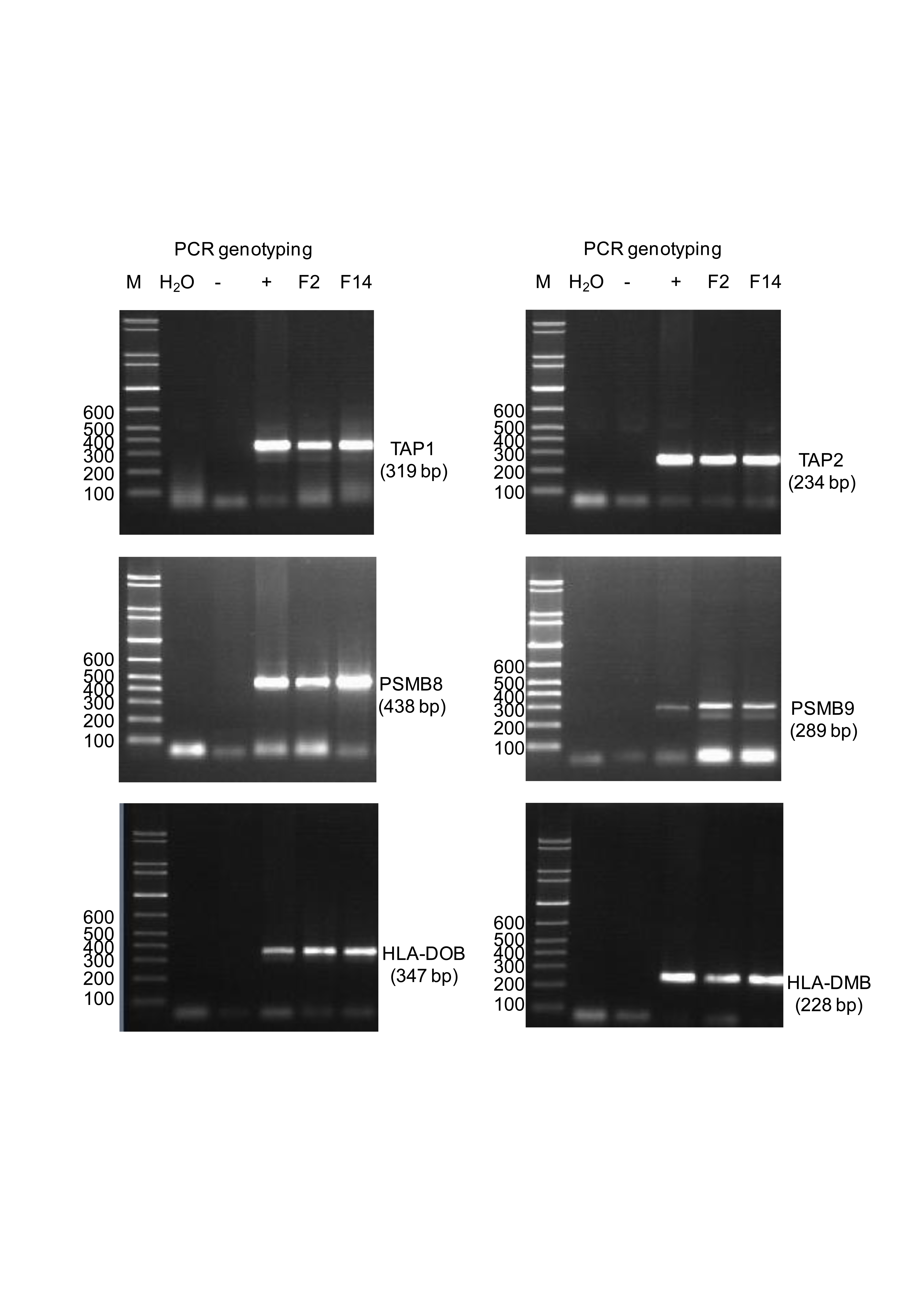


**Supplementary Figure 6. Full-length gels for PCR genotyping of human TAP1, TAP2, PSMB8, PSMB9, HLA-DOB and HLA-DMB in hTAP-LMP transgenic mice.** Indicated are markers with 100, 200, 300, 400, 500 and 600 bp, with size for each PCR product. F2 and F14 were the two positive founder mice. “-”, tail DNA samples of WT littermates; “+”, plasmid DNA of the BAC clone RP11-10A19.


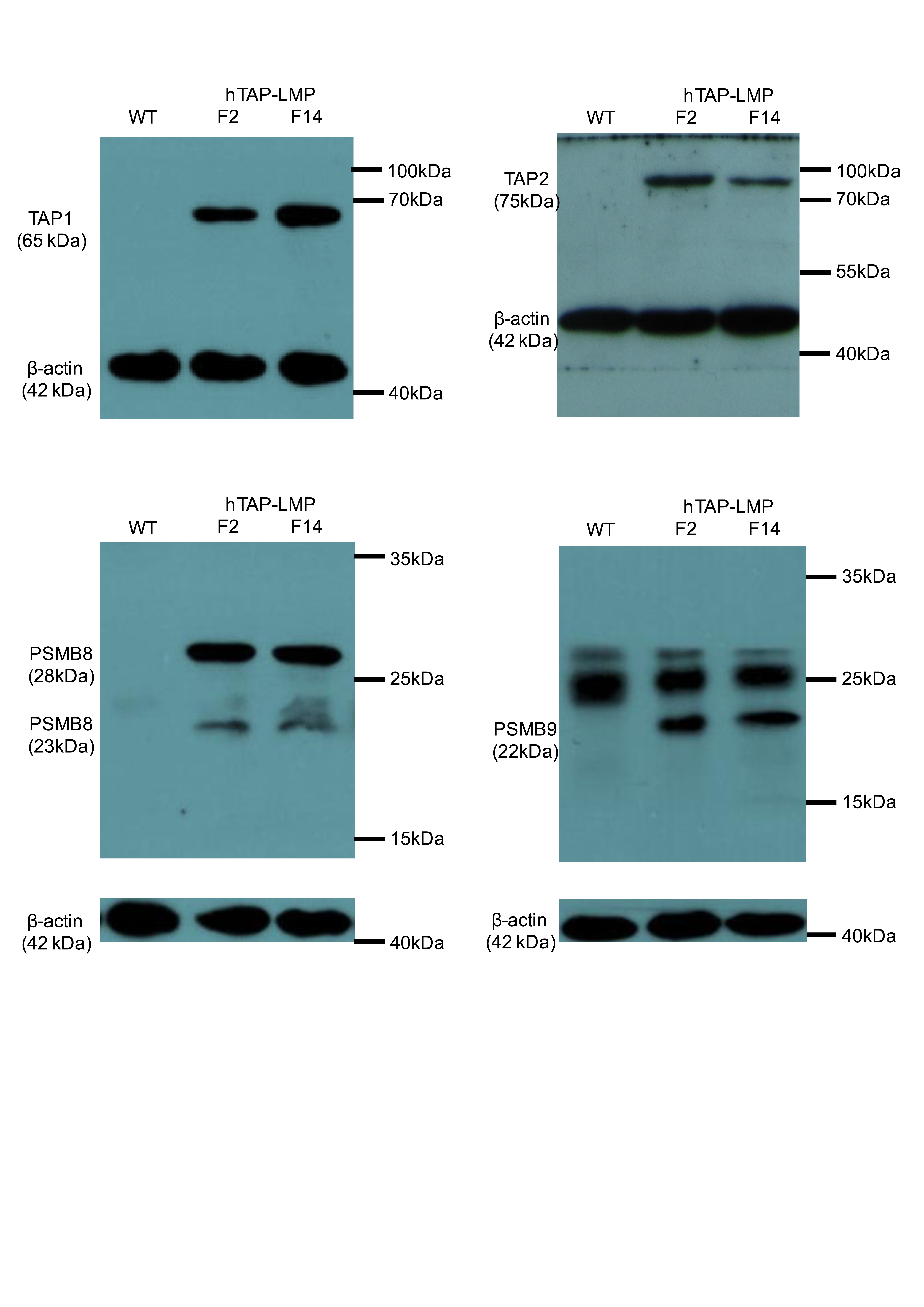


**Supplementary Figure 7. Full-length western blots for human TAP1, TAP2, PSMB8 and PSMB9 in hTAP-LMP transgenic mice.** Expression of human TAP1 (65 kDa), TAP2 (75 kDa), PSMB8 (23/28 kDa), and PSMB9 (22 kDa) in WT or hTAP-LMP mice was determined by western blotting; β-actin (42 kDa) was used as an internal control. The blot of PSMB9 has two bands near 25 kDa in both WT and hTAP-LMP mice, which probably represent a non-specific binding.
